# Overexpression of β1 integrin contributes to polarity reversal and a poor prognosis of breast invasive micropapillary carcinoma

**DOI:** 10.18632/oncotarget.22774

**Published:** 2017-11-30

**Authors:** Bingbing Liu, Xia Zheng, Fanfan Meng, Yunwei Han, Yawen Song, Fangfang Liu, Shuai Li, Lanjing Zhang, Feng Gu, Xinmin Zhang, Li Fu

**Affiliations:** ^1^ Department of Breast Cancer Pathology and Research Laboratory, Tianjin Medical University Cancer Institute and Hospital, National Clinical Research Center for Cancer, Key Laboratory of Cancer Prevention and Therapy, Tianjin, China; ^2^ Key Laboratory of Breast Cancer Prevention and Therapy, Tianjin Medical University, Ministry of Education, Tianjin, China; ^3^ Department of Pathology, Third Central Hospital of Tianjin, Tianjin Institute of Hepatobiliary Disease, Tianjin Key Laboratory of Artificial Cell, Artificial Cell Engineering Technology Research Center of Public Health Ministry, Tianjin, China; ^4^ Department of Pathology, University Medical Center of Princeton, Plainsboro, NJ, USA; ^5^ Rutgers Cancer Institute of New Jersey, New Brunswick, NJ, USA; ^6^ Department of Chemical Biology, Ernest Mario School of Pharmacy, Rutgers University, Piscataway, NJ, USA; ^7^ Department of Pathology, Robert Wood Johnson Medical School, and Cancer Institute of New Jersey, Rutgers University, New Brunswick, NJ, USA; ^8^ Department of Pathology, Cooper University Hospital, Cooper Medical School of Rowan University, Camden, NJ, USA

**Keywords:** invasive micropapillary carcinoma, polarity reversal, β1 integrin, metastasis, breast

## Abstract

Invasive micropapillary carcinoma (IMPC) of the breast is a highly aggressive breast cancer. Polarity reversal exemplified by cluster growth is hypothesized to contribute to the invasiveness and metastasis of IMPC. In this study, we demonstrate that levels of β1 integrin and Rac1 expression were greater in breast IMPC than in invasive breast carcinoma of no specific type and paraneoplastic benign breast tissue. We show that silencing β1 integrin expression using the β1 integrin inhibitor AIIB2 partially restored polarity in IMPC primary cell clusters and downregulated Rac1. Thus, overexpression of β1 integrin upregulates Rac1. Univariate analysis showed that overexpression of β1 integrin and Rac1 was associated with breast cancer cell polarity reversal, lymph node metastasis, and poor disease-free survival in IMPC patients. Multivariate analysis revealed that polarity reversal was an independent predictor of poor disease-free survival. These findings indicate that overexpression of β1 integrin and the resultant upregulation of Rac1 contribute to polarity reversal and metastasis of breast IMPC, and that β1 integrin and Rac1 could be potential prognostic biomarkers and targets for treatment of breast IMPC.

## INTRODUCTION

Invasive micropapillary carcinoma (IMPC) is a tumor with a propensity for metastasis that occurs in various organs [1–3]. It was first reported in the breast by Fisher *et al.* [4] in 1980, and the term was adopted by the World Health Organization (WHO) classification of breast tumors in 2003 [5]. We have previously shown that breast IMPC exhibits polarity reversal in cell clusters, which increases the risk of invasion and metastasis [6]. Polarity reversal can be identified by immunohistochemistry (IHC) for E-cadherin (E-cad), epithelial membrane antigen (EMA), mucin family protein-1 (MUC-1), and sialyl-Lewis X (SleX) [7–10]. Liu *et al.* [11] reported that presence of IMPC in breast mucinous carcinoma promotes tumor metastasis and that patients with mixed IMPC/mucinous carcinoma have worse recurrence-free survival and overall survival (OS) than patients with pure mucinous carcinoma. We theorized that polarity reversal of tumor cell clusters contributes to invasion and metastasis of IMPC and thus to its poor prognosis [12].

Integrins are a family of transmembrane receptors. They are heterodimers composed of α and β subunits. β1 integrin is mainly expressed in normal cells. Lee *et al.* [13] found that β1 integrin helps maintain polarity of normal epithelial cells and assists in the formation of glandular lumen. Aberrant expression of β1 integrin in human breast carcinoma has been linked to cell adhesion, angiogenesis, tumor progression, and metastasis [14, 15]. Overexpression of β1 integrin has been reported in several solid tumors [14, 16], and inhibition of β1 integrin expression in breast cancer cell lines restores the polarity of tumor cells to a status similar to normal mammary epithelial cells [17, 18]. Other studies showed that treatment of normal epithelial MDCK cells with β1 integrin inhibitor resulted in polarity disorder and malignant phenotype transformation [19, 20]. Thus, balanced expression of β1 integrin is required to maintain normal polarity.

Rac is a member of the Rho family of small GTPases that is regulated by integrin and affects a variety of actin-dependent processes including cell-cell adhesion, cell migration, and cellular transformation [21]. Studies have demonstrated that Rac1 induces epithelial polarity in cells adhering to extracellular matrix [22] and contributes to cell migration, loss of adhesion, invasion, and metastasis of tumors [23]. However, its expression and regulatory relationship with β1 integrin in IMPC have not been reported.

Here, we examined β1 integrin and Rac1 expression and assessed their effects on polarity at the cytologic level. We then validated our results in breast cancer cell lines and primary breast cancer cells. We further correlated our findings with patients’ clinical outcomes.

## RESULTS

### β1 integrin positively regulates Rac1 expression

**Figure 1:**
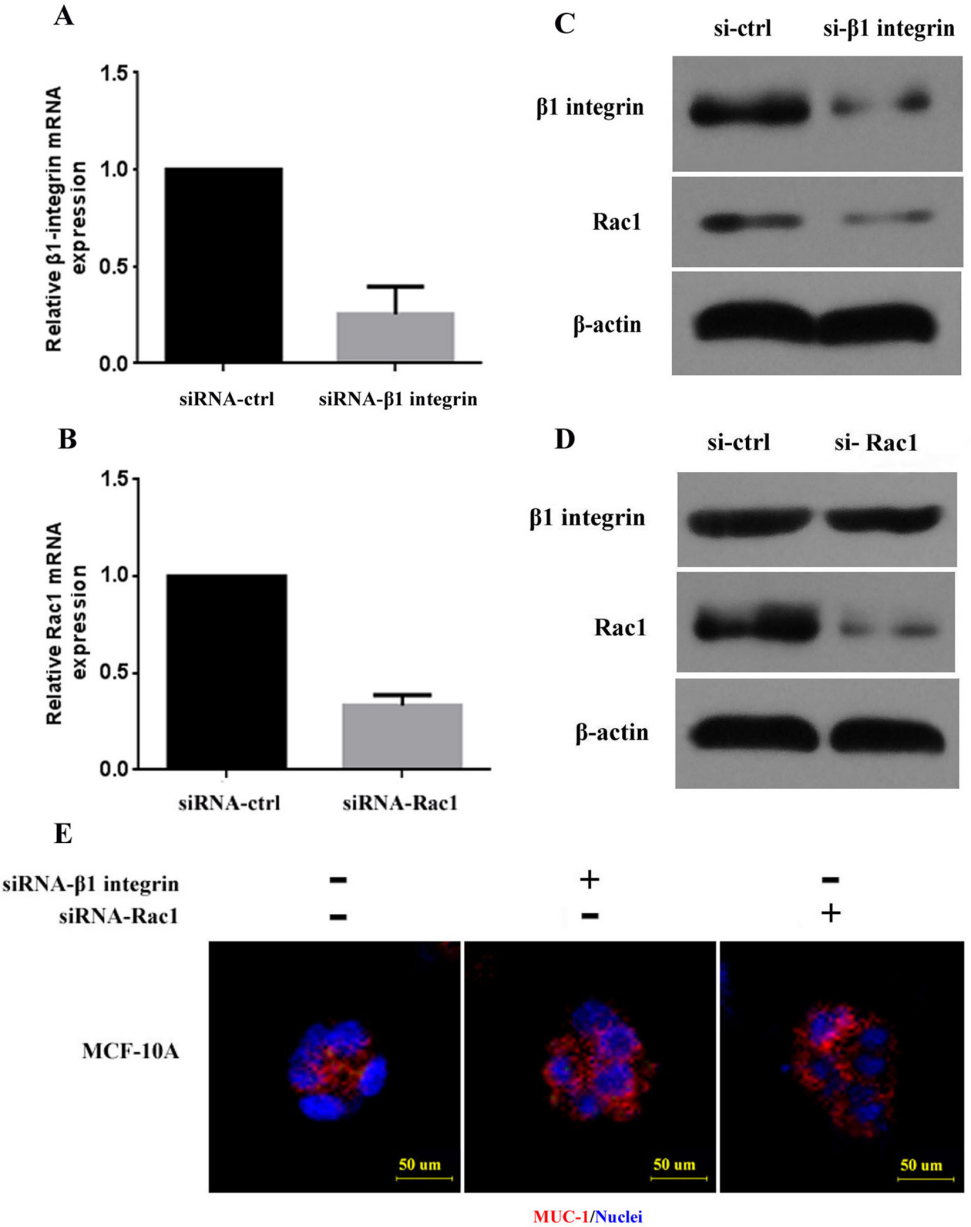
β1 integrin and Rac1 expression and polarity of breast cancer cell lines with silencing of β1 integrin and Rac1 (**A**, **B**) β1 integrin and Rac1 mRNA in MCF-10A was downregulated after transfection with siRNA-β1 integrin and siRNA-Rac1. (**C**, **D**) Decreased β1 integrin and Rac1 protein expression in MCF-10A was detected by Western blot after transfection with siRNA-β1 integrin and siRNA-Rac1. β-actin was used as control. (**E**) Disordered polarity of MCF-10A cell clusters in 3D culture after treatment with siRNA-β1 integrin and siRNA-Rac1 is shown. Normal polarity was determined by MUC-1 (red) at the luminal surface of control cells. Nuclei are shown with DAPI (blue). Scale bars, 50 μm. si-ctrl: control cell line.

We first evaluated the silencing effects of siRNA-β1 integrin and siRNA-Rac1 in MCF-10A normal breast epithelial cells. siRNA-ctrl was used as negative control. As shown in Figure 1A and 1B, β1 integrin mRNA was significantly decreased with siRNA-β1 integrin transfection. Rac1 mRNA was also significantly decreased with siRNA-Rac1 transfection. At the protein level, both β1 integrin expression and Rac1 expression were decreased (Figure 1C). When cells were transfected with siRNA-Rac1, Rac1 expression was downregulated, but no significant decrease in β1 integrin expression was noted (Figure 1D). The results indicate that Rac1 expression is positively regulated by β1 integrin.

### β1 integrin silencing leads to disordered polarity of MCF-10A cell clusters

To assess polarity changes induced by β1 integrin, we planted MCF-10A cells in collagen gel for three-dimensional (3D) culture and then silenced β1 integrin expression using siRNA. MUC-1, the marker of cell polarity, was detected by rhodamine-conjugated affinipure goat anti-rabbit IgG (red). Immunofluorescence analysis of the siRNA-ctrl control group showed that MUC-1 was expressed on the inner side of the cell clusters, indicating that MCF-10A cell clusters displayed normal polarity. As shown in Figure 1E, after treatment with siRNA-β1 integrin, MUC-1 staining was predominantly located on the stroma-facing surface of the cell clusters, demonstrating polarity reversal. The cell clusters were transformed from having a hollow growth pattern to irregular clusters. When cells were treated with siRNA-Rac1, cell cluster polarity also became disordered (Figure 1E). These observations indicate that loss of β1 integrin and/or Rac1 can lead to disordered cell polarity in 3D culture.

### AIIB2 downregulates Rac1 in breast cancer cell lines and IMPC primary tumor cells

**Figure 2:**
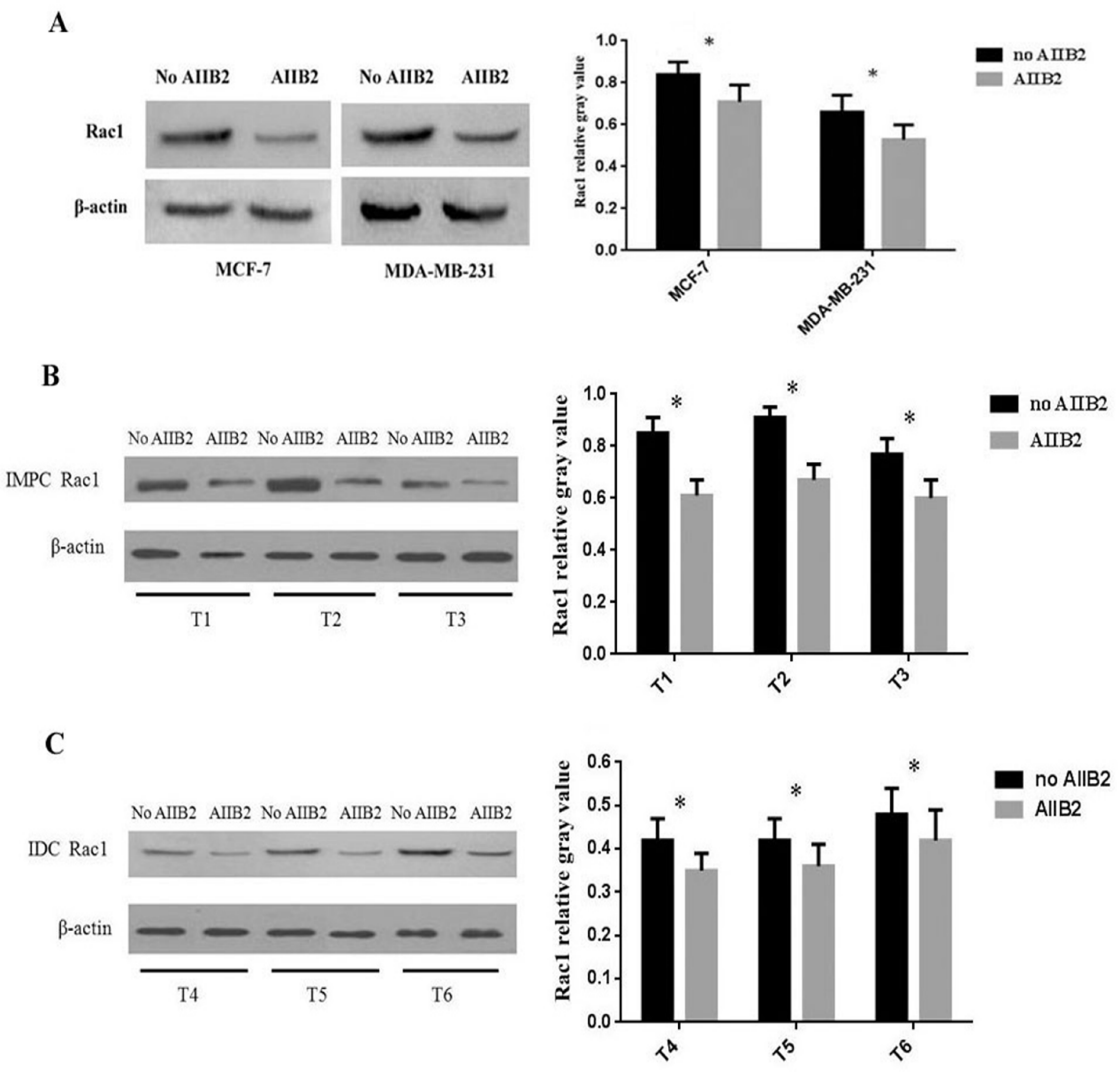
Expression of β1 integrin and Rac1 in breast cancer cell lines and primary tumor cells at 3D culture treated with β1 integrin inhibitor AIIB2 and detected by Western blot (**A**) Rac1 expression in MCF-7 and MDA-MB-231 was lower than in controls. (**B**, **C**) Rac1 expression in IMPC and IDC-NST tumor cells was decreased. β-Actin was used as control. Relative gray value was defined as the ratio between the gray value of Rac1 and β-actin (student’s *t*-test, ^*^P < 0.05). T1-T3: 3 cases of IMPC primary tumor; T4-T6: 3 cases of IDC-NST primary tumor.

In 3D cell culture, we treated MCF-7 and MDA-MB-231 breast cancer cell lines, as well as primary tumor cells of IMPC and invasive ductal carcinoma of no specific type (IDC-NST), with AIIB2, a β1 integrin inhibitor used in previous studies [24–26]. After 72 hours of incubation with AIIB2, Rac1 protein levels decreased in both cancer cell lines (Figure 2A) and primary tumor cells (Figure 2B, 2C), indicating that AIIB2-induced inhibition of β1 integrin downregulates Rac1. These findings support the earlier conclusion that β1 integrin positively regulates Rac1 expression.

### AIIB2 causes polarity change in IMPC cell clusters

**Figure 3:**
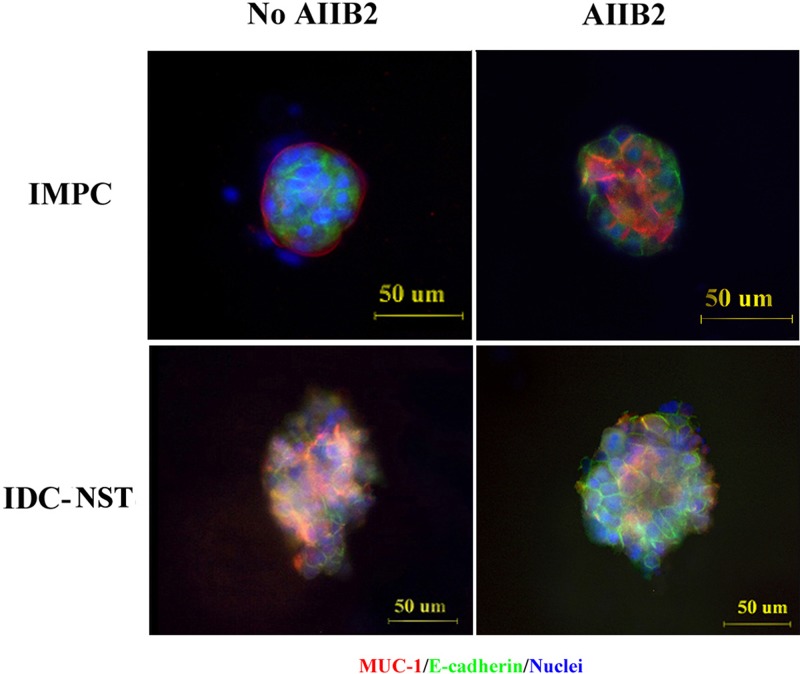
Polarity alterations induced by the β1 integrin inhibitor AIIB2 IMPC and IDC-NST cells were stained with anti-MUC-1 antibody (red) and anti-E-cadherin antibody (green), and the nuclei were stained with DAPI (blue). Representative confocal images were taken. Scale bars, 50 μm.

To assess whether β1 integrin induces polarity changes, we planted IMPC primary tumor cells in collagen gel for 3D culture and then treated the cells with AIIB2. Rhodamine-conjugated affinipure goat anti-rabbit IgG was used to label MUC-1 (red), and rhodamine-conjugated affinipure goat anti-mouse IgG was used to label E-cad (green). Immunofluorescence analysis identified polarity reversal in IMPC cell clusters without AIIB2 treatment. After AIIB2 treatment, polarity was partially restored. Similar changes were observed in IDC-NST cells (Figure 3). These observations suggest that β1 integrin overexpression causes tumor cell polarity reversal.

### β1 integrin and Rac1 are overexpressed in IMPC

**Figure 4:**
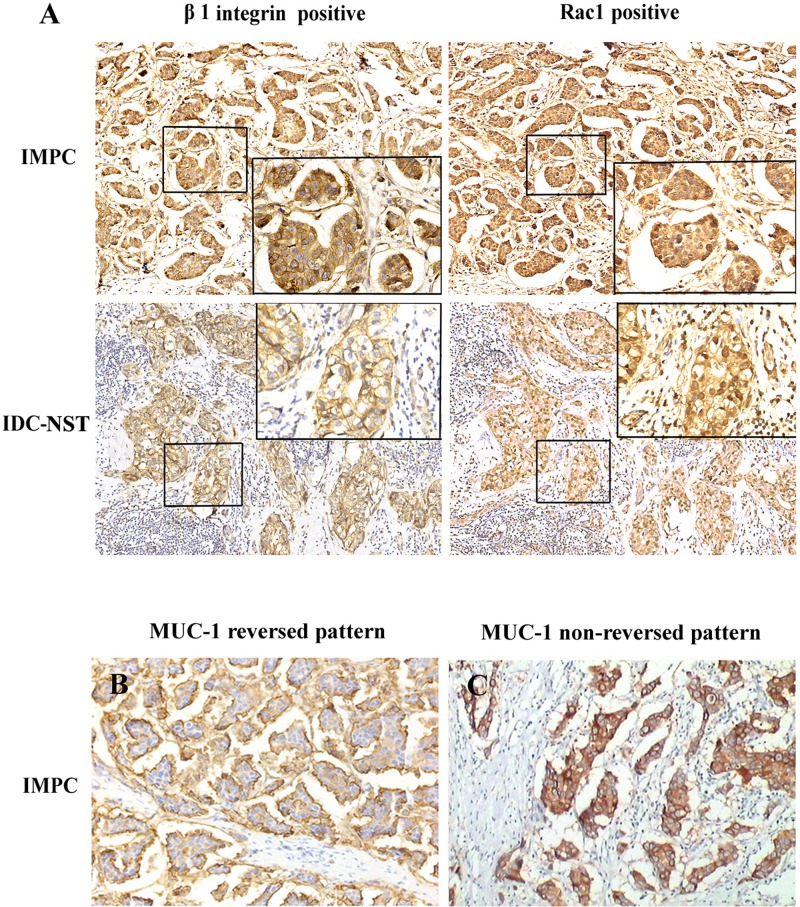
(A) Immunohistochemical stain of β1 integrin and Rac1 in IMPC and IDC-NST (×200) β1 integrin was predominantly expressed on the cell membrane with brown-yellow color, whereas Rac1 was primarily expressed in the nucleus and cytoplasm. (B) Reversed immunohistochemistry pattern of MUC-1 in IMPC, which was defined as the presence of complete linear reactivity on the outer surface of tumor cell clusters facing stroma (×200). (C) Nonreversed immunohistochemistry pattern of MUC-1 in IMPC, which was defined as whole cytoplasmic membrane staining of tumor cell clusters (×200).

We evaluated β1 integrin and Rac1 expression in breast IMPC tissues by IHC. β1 integrin was mainly expressed on the cell membrane with brown-yellow color (Figure 4A). Rac1 was predominantly expressed in the nucleus, whereas the cytoplasm was stained with brown particles (Figure 4A). Of 102 IMPC samples, 80 (78.4%) demonstrated high β1 integrin levels and 81 (79.4%) demonstrated high Rac1 levels. Of 48 paraneoplastic benign breast tissue samples, 30 (62.5%) demonstrated high β1 integrin levels and 29 (60.4%) demonstrated high Rac1 levels. β1 integrin and Rac1 levels were significantly greater in IMPC than paraneoplastic benign breast tissue (*P* = 0.040 and *P* = 0.035, respectively) (Table 1).

**Table 1:** β1 integrin and Rac1 expression in IMPC and paraneoplastic benign breast tissue

Protein expression	Paraneoplastic benign breast *n* (%)	IMPC *n* (%)	χ^2^	*P* value^*^
β1 integrin				
High	30 (62.5)	80 (78.4)	4.236	0.040
Low	18 (37.5)	22 (21.6)		
Rac1				
High	29 (60.4)	81 (79.4)	4.451	0.035
Low	17 (35.4)	21 (20.6)		

IMPC:invasive micropapillary carcinoma.

^*^P values were calculated by χ^2^ test.

Of 100 IDC-NST samples, 63 (63%) demonstrated high β1 integrin levels and 67 demonstrated high Rac1 levels. β1 integrin and Rac1 levels were significantly greater in IMPC than IDC-NST (*Z* = –2.571, *P* = 0.010; and *Z* = –1.988, *P* = 0.047 respectively) (Table 2).

**Table 2:** Clinicopathological characteristics of IMPC and IDC-NST patients

Characteristics	IMPC(%)	IDC-NST(%)	*Z*	*P* value^*^
Age (years)				
≤52	43 (42.2)	55 (55)	−1.882	0.069
>52	59 (57.8)	45 (45)		
Tumor size (T stage)				
T1	28 (27.5)	36 (36)	−1.712	0.087
T2	60 (58.8)	57 (57)		
T3–T4	14 (13.7)	7 (7)		
Histological grade				
I	10 (9.8)	13 (13)	−1.512	0.131
II	67 (65.7)	71 (71)		
III	25 (24.5)	16 (16)		
Lymph node status				
No metastasis	18 (17.6)	47 (47)	−4.454	0.000
metastasis	84 (82.4)	53 (53)		
ER status				
Negative	43 (42.2)	41 (41)	−0.166	0.868
Positive	59 (57.8)	59 (59)		
PR status				
Negative	41 (40.2)	39 (39)	−0.173	0.862
Positive	61 (59.8)	61 (61)		
Her2 status				
Negative	83 (81.4)	71 (71)	−1.727	0.084
Positive	19 (18.6)	29 (29)		
β1 integrin expression				
Low	21 (20.6)	37 (37)	−2.571	0.010
High	81 (79.4)	63 (63)		
Rac1 expression				
Low	21 (20.6)	33 (33)	−1.988	0.047
High	81 (79.4)	67 (67)		
MUC-1 expression				
Non-reversed pattern	15 (14.7)	96 (96)	−11.582	0.000
Reversed pattern	87 (85.3)	4 (4)		

ER:estrogen receptor, PR:progesterone receptor, HER-2:human epidermal growth factor receptor 2, IMPC:invasive micropapillary carcinoma, IDC-NST: invasive breast carcinoma of no specific type.

^*^P values were calculated by Mann-Whitney *U* test.

### Upregulated β1 integrin expression correlates with lymph node metastasis (LNM), polarity reversal, and Rac1 overexpression in IMPC

IMPC patients with high β1 integrin expression had a higher incidence of LNM than patients with low expression (87.7% vs 61.9%; *P* = 0.006). Similarly, IDC-NST patients with high β1 integrin expression had a higher incidence of LNM than patients with low expression (61.9% vs 37.8%; *P* = 0.020) (Table 3). Overexpression of β1 integrin in IMPC was positively correlated with LNM (rs = 0.273, *P* = 0.005), Rac1 expression (rs = 0.280, *P* = 0.004), and polarity reversal (rs = 0.268, *P* = 0.007). However, no significant association was observed between β1 integrin expression and age, tumor size, histologic grade, or estrogen receptor (ER), progesterone receptor (PR), or human epidermal growth factor receptor 2 (HER2) status (Table 4).

**Table 3:** Lymph node metastasis of IMPC and IDC-NST with low or high expression of β1 integrin and Rac1

Histologycal type	LN status	β1 integrin expression		χ^2^	P^*^ value	Rac1 expression		χ^2^	P^*^ value
		high	low			high	low		
IMPC									
	Metastasis	71	13	7.608	0.006	71	12	11.565	0.001
	No metastasis	10	8			9	9		
IDC-NST									
	Metastasis	39	14	5.420	0.020	41	12	5.472	0.019
	No metastasis	24	23			26	21		

IMPC:invasive micropapillary carcinoma, IDC-NST: invasive breast carcinoma of no specific type, LN: Lymph node

^*^P values were calculated by χ^2^ test.

**Table 4:** β1 integrin expression and clinicopathological characteristics of IMPC patients

Characteristics	β1 integrin expression		r_s_	*P* value^*^
	Low	High		
Age(years)				
≤52	6	37	−0.140	0.160
>52	15	44		
Tumor size (T stage)				
T1	3	25	−0.014	0.889
T2	18	42		
T3–T4	0	14		
Histological grade				
I	2	8	0.095	0.343
II	16	51		
III	3	22		
Lymph node status				
No metastasis	8	10	0.273	0.005
metastasis	13	71		
ER status				
Negative	5	38	–0.189	0.057
Positive	16	43		
PR status				
Negative	5	36	−0.170	0.087
Positive	16	45		
Her2 status				
Negative	18	65	0.057	0.571
Positive	3	16		
Rac1 expression				
Low	9	12	0.280	0.004
High	12	69		
MUC-1 expression				
Non-reversed pattern	7	8	0.268	0.007
Reversed pattern	14	73		

ER:estrogen receptor, PR:progesterone receptor, HER-2:human epidermal growth factor. receptor 2, IMPC:invasive micropapillary carcinoma.

^*^P values were calculated by Spearman's Rank-Correlation test.

### Upregulated Rac1 expression correlates with LNM and polarity reversal in IMPC

IMPC patients with high Rac1 expression had a higher incidence of LNM than patients with low expression (88.9% vs 57.1%; *P* = 0.001). Similarly, IDC-NST patients with high Rac1 expression had a higher incidence of LNM than patients with low expression (61.2% vs 36.3%; *P* = 0.019) (Table 3). Overexpression of Rac1 in IMPC was positively correlated with LNM (rs = 0.337, *P* = 0.001), β1 integrin expression (rs = 0.280, *P* = 0.004), and polarity reversal (rs = 0.268, *P* = 0.007). No significant associations were observed between Rac1 expression and age, tumor size, histologic grade, or ER, PR, or HER2 status (Table 5).

**Table 5:** Rac1 expression and clinicopathological characteristics of IMPC patients

Characteristics	Rac1 expression		r_s_	*P* value^*^
	Low	High		
Age(years)				
≤52	9	34	0.007	0.943
>52	12	47		
Tumor size (T stage)				
T1	5	23	–0.001	0.993
T2	14	46		
T3-T4	2	12		
Histological grade				
I	3	7	0.042	0.673
II	13	54		
III	5	20		
Lymph node status				
No metastasis	9	9	0.337	0.001
metastasis	12	72		
ER status				
Negative	9	34	0.007	0.943
Positive	12	47		
PR status				
Negative	5	36	–0.170	0.087
Positive	16	45		
Her2 status				
Negative	16	67	–0.068	0.499
Positive	5	14		
β1 integrin expression				
Low	9	12	0.280	0.004
High	12	69		
MUC-1 expression				
Non-reversed pattern	7	8	0.268	0.007
Reversed pattern	14	73		

ER:estrogen receptor, PR:progesterone receptor, HER-2:human epidermal growth factor. receptor 2, IMPC:invasive micropapillary carcinoma.

^*^P values were calculated by Spearman's Rank-Correlation test.

LNM rate is significantly higher for IMPC and IDC-NST tumors with weak β1 integrin and Rac1 expression than in tumors with negative expression

Twenty-one IMPCs showed low β1 integrin expression: 14 had weak expression and 7 had negative expression. Eleven (78.6%) of 14 IMPC patients with weak β1 integrin expression had LNM, whereas 2 (28.6%) of 7 patients with negative expression had LNM (*P* = 0.026) (Table 6). Thirty-seven IDC-NSTs showed low β1 integrin expression: 21 had weak expression and 16 had negative expression. Eleven (52.4%) of 21 IDC-NST patients with weak β1 integrin expression had LNM, whereas 3 (18.8%) of 16 patients with negative expression had LNM (*P* = 0.037) (Table 6).

**Table 6:** Lymph node metastasis in IMPC and IDC-NST with β1 integrin and Rac1 low expression

Pathology type	LN status	β1 integrin expression		χ^2^	*P* value^*^	Rac 1 expression		χ^2^	*P* value^*^
		+	–			+	–		
IMPC	LN+	11	2	4.947	0.026	10	2	5.452	0.020
	LN−	3	5			3	6		
IDC-NST	LN+	11	3	4.367	0.037	8	4	4.537	0.033
	LN−	10	13			6	15		

IMPC:invasive micropapillary carcinoma, IDC-NST: invasive breast carcinoma of no specific type.

^*^P values were calculated by χ^2^ test.

Twenty-one IMPCs showed low Rac1 expression: 13 had weak expression and 8 had negative expression. Ten (76.9%) of 13 IMPC patients with weak Rac1 expression had LNM, whereas 2 (25%) of 8 patients with negative expression had LNM (*P* = 0.020) (Table 6). Thirty-three IDC-NSTs showed low Rac1 expression: 14 had weak expression and 19 had negative expression. Eight (57.1%) of 14 IDC-NST patients with weak Rac1 expression had LNM, whereas 4 (21.1%) of 19 patients with negative expression had LNM (*P* = 0.033) (Table 6).

### Polarity reversal correlates with LNM and β1 integrin expression in IMPC patients

Among the 102 IMPC patients, 84 (82.4%) demonstrated LNM, an incidence significantly higher than that in IDC-NST patients (53%; *P* = 0.000) (Table 2). No significant differences in patient age, tumor size, histologic grade, or ER, PR, or HER2 status were identified between the IMPC and IDC-NST groups (*P* > 0.05) (Table 2).

We next investigated the association between polarity reversal (Figure 4B, 4C) and β1 integrin expression, Rac1 expression, and LNM rate *in vivo* by MUC-1 IHC. Among the 102 IMPC samples, 87 (85.3%) showed reversed polarity; in contrast, 4 (4%) of 100 IDC-NST samples showed reversed polarity (Table 2). Polarity reversal in IMPC was positively correlated with overexpression of β1 integrin (rs = 0.268, *P* = 0.007) and Rac1 (rs = 0.268, *P* = 0.007) and presence of LNM (rs = 0.316, *P* = 0.001). No significant association was observed between polarity reversal and age, tumor size, histologic grade, or ER, PR, or HER2 status (*P* > 0.05) (Table 7). Our results indicate that polarity reversal is associated with increased LNM in IMPC.

**Table 7:** MUC-1 expression and clinicopathological parameters of IMPC patients

Characteristics	MUC-1 expression		r_s_	*P* value^*^
	Non-reversed pattern	Reversed pattern		
Age (years)				
≤52	4	39	–0.130	0.192
>52	11	48		
Tumor size (T stage)				
T1	4	24	–0.003	0.974
T2	9	51		
T3–T4	2	12		
Histological grade				
I	3	7	0.101	0.312
II	9	58		
III	3	22		
Lymph node status				
No metastasis	7	11	0.316	0.001
metastasis	8	76		
ER status				
Negative	5	38	–0.074	0.459
Positive	10	49		
PR status				
Negative	3	38	–0.171	0.086
Positive	12	49		
Her2 status				
Negative	12	71	0.015	0.884
Positive	3	16		
β1 integrin expression				
Low	7	14	0.268	0.007
High	8	73		
Rac1 expression				
Low	7	14	0.268	0.007
High	8	73		

ER:estrogen receptor, PR:progesterone receptor, HER-2:human epidermal growth factor. receptor 2, IMPC:invasive micropapillary carcinoma.

^*^P values were calculated by Spearman's Rank-Correlation test.

### Overexpression of β1 integrin correlates with poor prognosis of IMPC patients

**Figure 5:**
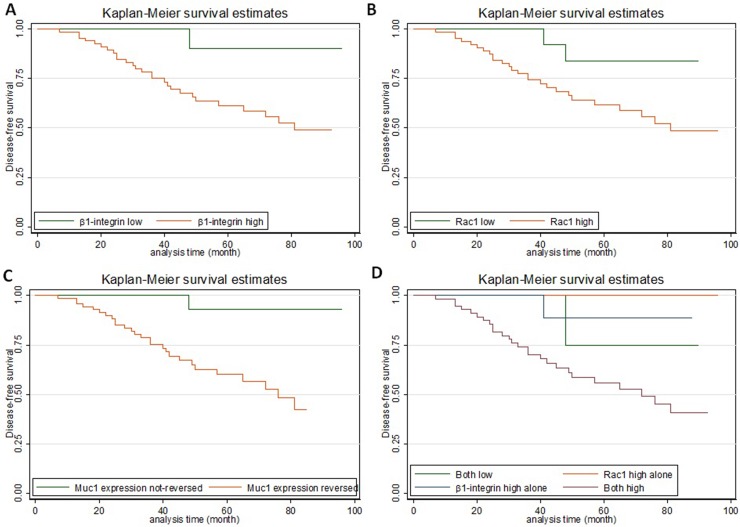
Kaplan-Meier curves showing the prognostic value of β1 integrin, Rac1, and (reversal of) MUC-1 expression in IMPC (**A**) High β1 integrin expression was correlated with shorter disease-free survival (DFS) (*P* = 0.017). (**B**) High Rac1expression was correlated with shorter DFS (*P* = 0.027). (**C**) MUC-1 reversed pattern was correlated with shorter DFS (*P* = 0.004). (**D**) The DFS of patients with high β1 integrin and Rac1 expression was shorter than that of patients with high expression of only one of these markers (both high vs β1 integrin high, *P* = 0.029; both high vs Rac1 high, *P* = 0.019; and both high vs both low, *P* = 0.15).

To assess whether β1 integrin expression correlates with the prognosis of IMPC patients, DFS and OS analyses were performed in all patients. Kaplan-Meier survival curves showed that DFS was significantly shorter in patients with overexpression of β1 integrin or Rac1 or with polarity reversal than in patients with low β1 integrin or Rac1 expression or without polarity reversal (*P* = 0.017, *P* = 0.026, and *P* = 0.003, respectively) (Figure 5A–5C). The DFS of patients with both β1 integrin and Rac1 overexpression was significantly shorter than in patients with overexpression of only β1 integrin or Rac1 (*P* = 0.029 and *P* = 0.019 separately), whereas no significant difference in DFS was noted between patients with overexpression of both β1 integrin and Rac1 and patients with low expression of both of these markers (Figure 5D). No significant OS differences were observed between patients with and without high expression of β1 integrin and/or Rac1 or patients with and without polarity reversal (data not shown). Univariate Cox regression analysis revealed that increased tumor size, presence of LNM, overexpression of β1 integrin and/or Rac1, and polarity reversal are associated with worse DFS in patients with IMPC (*P* < 0.05) (Table 8). However, multivariate Cox regression analysis only confirmed that increased tumor size (*P* = 0.010) and polarity reversal (*P* = 0.031) were independent predictors of poor DFS in IMPC patients (Table 8).

**Table 8:** Univariate and multivariate cox regression analysis for disease-free survival of breast IMPC patients

Variables	Univariable			Multivariable		
	HR	95% CI	*P* value	HR	95% CI	*P* value
Age (years)						
≤52 vs >52	0.835	0.395–1.769	0.639	–	–	–
Tumor size (T stage)						
T1 vs T2 vs T3–T4	1.856	1.036–3.327	0.038	2.091	1.166–3.749	0.013
Histological grade						
I vs II vs III	1.043	0.517–2.105	0.907	–	–	–
Lymph node status						
No metastasis vs metastasis	9.080	1.227–67.192	0.031	3.521	0.459–27.032	0.226
ER status						
Negative vs Positive	0.922	0.436–1.950	0.832	–	–	–
PR status						
Negative vs Positive	0.701	0.333–1.475	0.349	–	–	–
Her2 status						
Negative vs Positive	0.980	0.396–2.426	0.965	–	–	–
β1 integrin expression						
Low vs High	7.806	1.060–57.479	0.044	2.913	0.379–22.379	0.304
Rac1 expression						
Low vs High	4.446	1.054–18.754	0.042	3.005	0.666–13.553	0.152
MUC-1 expression						
Non-reversed pattern vs Reversed pattern	11.658	1.543–88.061	0.017	8.005	1.025–62.521	0.047

ER: estrogen receptor, PR: progesterone receptor, HER-2: human epidermal growth factor receptor 2, IMPC: invasive micropapillary carcinoma, HR: hazard ratio, CI: confidence interval.

## DISCUSSION

Pure breast IMPC is rare, constituting <2% of breast cancers; however, IMPC has a high rate of metastasis. Currently, there are no universally accepted criteria for pathologic diagnosis of IMPC, particularly regarding the required proportion of micropapillary clusters in a nonhomogeneous tumor. In this study, we selected tumors consisting of at least 50% IMPC component. Based on previous studies, we hypothesized that tumor cell polarity reversal is a critical factor facilitating metastasis. The integrins constitute a family of polarity-related proteins, and their upregulation in breast cancer cells has been reported [27], although conflicting results have also been described [28]. We chose to study the expression of β1 integrin and Rac1 in breast cancer cell lines and IMPC and correlated our findings with the clinicopathologic features of patients. To our knowledge, this is the first study to investigate the role of β1 integrin and Rac1 expression in IMPC.

We showed that when β1 integrin expression was blocked in the MCF-10A cell line by siRNA-β1 integrin, the expression of Rac1 was also significantly downregulated. In contrast, when Rac1 expression was blocked in the same cell line by siRNA-Rac1, no significant decrease in β1 integrin expression was found. The results confirm that, at least in the MCF-10A cell line, β1 integrin positively regulates Rac1 expression, but not the reverse, and indicate that Rac1 is in a downstream position to β1 integrin in the chain of molecular processes that maintain epithelial cell polarity.

Using 3D culture, we demonstrated that the polarity of MCF-10A clusters became disordered after treatment with siRNA-β1 integrin; the cell clusters were transformed from normal clusters with a hollow growth pattern to irregular cell clusters, as reported previously by others [19, 20]. Weaver *et al.* [17] reported that treatment of breast cancer cells with inhibitory β1 integrin antibody leads to a striking morphologic and functional reversion to a normal phenotype. We confirmed these results in our study, in which treatment with the β1 integrin inhibitor AIIB2 partially abrogated the polarity reversal seen in IMPC clusters. These results indicate that upregulation of β1 integrin causes polarity reversal of IMPC cells, followed by formation of tumor cell clusters as a result of upregulation of Rac1. This could explain how morula-like clusters of IMPC are formed and could support our previous findings that IMPC tumor cells divorce from primary tumor and then invade and metastasize through tumor cell clusters [12].

Our immunohistochemical analysis revealed that β1 integrin and Rac1 expression was higher in IMPC than in paraneoplastic benign breast tissue and IDC-NST. IMPC patients with high expression of both β1 integrin and Rac1 had a significantly higher incidence of LNM than patients with low expression of both markers or with high expression of only one marker. In patients with IMPC that showed weak expression of β1 integrin and/or Rac1, the incidence of LNM was significantly greater than in patients with no expression. A similar trend was observed in patients with IDC-NST. A study in patients with IDC-NST showed that increased Rac1 expression was associated with partial reversed cell polarity and LNM, which supports the idea that IDC-NST with partial reversed cell polarity may be part of the IMPC spectrum [8, 29]. Univariate analysis showed that β1 integrin and Rac1 overexpression was associated with tumor cell polarity reversal, presence of LNM, and decreased DFS in IMPC patients. Multivariate analysis indicated that polarity reversal was an independent predictor for poor DFS in IMPC patients. These results suggest that upregulated β1 integrin may promote LNM of IMPC through formation of tumor clusters with polarity reversal. Perhaps this proposed model could be applied to other metastasis-prone tumors with IMPC growth patterns, including tumors of the lungs, ovaries, and colon [1–3].

Of note, patients with IMPC with high expression of both β1 integrin and Rac1 had a worse DFS than patients with high expression of either β1 integrin or Rac1. One explanation for this result is that tumor cell polarity reversal might require Rac1 to be activated by β1 integrin. Both β1 integrin and Rac1 could be involved in multiple molecular pathways, and activation of Rac1 by other unknown factors might lead to unrelated biologic events. This might explain why high expression of Rac1 alone had virtually no impact on DFS and why high expression of β1 integrin alone had minimal impact (Figure 5D). In addition, the prognostic difference between high expression of both markers and low expression of both markers did not reach statistical significance. However, the number of IMPC patients included in the clinical assessment was small, and it is possible that low expression of both β1 integrin and Rac1 represents another abnormality unrelated to polarity reversal. Investigations using greater numbers of patients are required to validate these findings.

In conclusion, this study indicates that that high metastatic potential of breast IMPC is associated with polarity reversal of tumor cell clusters. β1 integrin positively regulates Rac1, an important factor involved in polarity reversal in IMPC. Overexpression of β1 integrin and the associated upregulation of Rac1 are associated with cancer cell polarity reversal, presence of LNM, and poor DFS, and polarity reversal is an independent predictor of poor prognosis of breast IMPC patients. Additional studies are warranted to validate these findings and to further explore the mechanisms involved in tumor growth and metastasis of breast IMPC.

## MATERIALS AND METHODS

Antibodies used include anti-β1 integrin (ab52971, Abcam), anti-Rac1 (ab33186, Abcam), anti-E-cad (ab1416, Abcam), and anti-MUC-1 (ab109185, Abcam). Other reagents included β1 integrin inhibitor AIIB2 (Developmental Studies Hybridoma Bank, United States), type I collagenase (Solarbio, China), collagen gel (Nitta Gelatin, Japan), culture media (Gibco), and Lipofectamine 2000 and TRIzol (Invitrogen). siRNAs were synthesized by GenePharma, China, and the sequences were as follows: siRNA-β1 integrin: forward, 5′-GUU UAA UGU CUG GUG CUU TT-3′; reverse, 5′-AAG CAC CAG ACA UUA AAC TT-3′; siRNA-Rac1: forward, 5′-UAA AGA CAC GAU CGA GAA AUU-3′; reverse, 5′-UUU CUC GAU CGU GUC UUU AUU-3′; and siRNA-ctrl: forward, 5′-UUC UCC GAA CGU GUC ACG UTT-3′; reverse, 5′-ACGUGACACGUUCGGAGAATT-3′.

### Breast cancer tissue samples

Formalin-fixed, paraffin-embedded tissue blocks from 102 patients with breast IMPC collected from 2007 to 2008 were retrieved from the archive of the Department of Breast Cancer Pathology and Research Laboratory, Tianjin Medical University Cancer Hospital, Tianjin, China. All cases had an IMPC component constituting >50% of the tumor volume. Paraneoplastic benign breast tissue was available in 48 blocks. In addition, 100 random cases of IDC-NST were retrieved during the same period as the control group. The diagnosis was confirmed independently by two pathologists using World Health Organization (WHO) criteria [30]. None of the patients received preoperative radiation or chemotherapy. Patients were followed for 1 to 100 months, with a median follow-up time of 63 months. Fresh tumor tissue samples were obtained from five IMPC and five IDC-NST patients who underwent surgical resection from November 2015 to May 2016. The research protocol was approved by the Tianjin Medical University Institutional Review Board, and informed consent was obtained from all participants.

### Cell culture

MCF-7 and MDA-MB-231 cell lines were grown in DMEM high glucose supplemented with 10% fetal bovine serum and 1% penicillin-streptomycin (P/S) solution at 37°C in a 5% CO2 incubator. The MCF-10A cell line was grown in DMEM/F12 supplemented with 5% horse serum, 10 μg/mL of insulin, 20 ng/mL of epidermal growth factor, 0.5 μg/mL of hydrocortisone, 1% NEAA, and 1% P/S solution at 37°C in a 5% CO2 incubator.

In breast cancer primary culture, after removal of adipose tissue, tumor tissue was treated as previously described [31]. Cells cultured in flask were then digested and centrifuged. Collagen gel was prepared on ice for 3D cell culture. The cells were counted and embedded in collagen gel with a cell density of 1.5 × 10^5^/mL. The collagen gel was then planted in a 24-well plate. After incubation at 37°C for 1 hour, complete medium was added and changed in 3-day intervals. After incubation for 3 to 7 days, spheroids of various sizes were harvested. For the inhibitor assessment, AIIB2 (7.5 μg/mL) was added on the day of planting, and the medium was replaced with a new one containing fresh inhibitor every 3 days. Control cultures were treated with ordinary medium only.

### siRNA transfection

Cells were treated with siRNA and Lipofectamine 2000. After 6 hours of incubation, transfection medium was replaced with complete culture medium for 48 hours. To verify the siRNA efficacy, cells were lysed and β1 integrin and Rac1 expression was analyzed by real-time polymerase chain reaction (PCR) and Western blot.

### Real-time PCR

TRIzol reagent was used to isolate RNA from cultured cells. cDNA was synthesized by SuperScript III reverse transcriptase (Invitrogen) using random primers. PCR reactions were performed using QuantiTect SYBR Green RT-PCR Kit (Qiagen), according to the manufacturer's instructions. The sequences of the semiquantitative reverse transcriptase PCR primers were as follows: β1 integrin: forward 5′-CAT CTG CGA GTG TGG TGT CT-3′, reverse 5′-GGG GTA ATT TGT CCC GAC TT-3′; Rac1: forward 5′-AGC TTT TGC GGA GAT TTT GA-3′, reverse 5′-CCC GTG ACA CTT TCA TTC CT-3′; and GAPDH: forward 5′-CGG AGT CAA CGG ATT TGG TCG TAT-3′, reverse 5′-AGC CTT CTC CAT GGT GGT GAA GAC-3′. Initial denaturation was at 94°C for 2 minutes, followed by 30 cycles of 94°C for 20 seconds and 60° for 30 seconds.

### Western blot

Three-dimensional cultured collagen gel was digested by type I collagenase and centrifuged before protein extraction. Total protein extracts from 3D cell cultures were prepared as described previously [24]. The protein concentration of each lysate was measured using the BCA method (Beyotime, China). Twenty micrograms of protein from each sample were separated by 10% SDS-polyacrylamide gel electrophoresis and transferred to PVDF membranes (Millipore, Billerica, MA) for 2 hours. The membranes were blocked with 5% skim milk for 1 hour. After overnight incubation with primary antibody β1 integrin (1:5000) and Rac1 (1:5000), membranes were incubated with secondary antibodies for 1 hour, and proteins were detected using the LiCor Odyssey Infrared Imaging System. Anti-β-actin antibodies were used as internal control.

### Immunofluorescence analysis

Primary tumor cells in 3D culture were fixed with 4% paraformaldehyde for 20 minutes and washed three times with phosphate-buffered saline (PBS); then 0.25% Triton X-100 (PBS prepared) was added for 20 minutes. The collagen gel was blocked with 3% BSA for 40 minutes. The cells were then washed with PBS and incubated with primary antibodies E-cad (1:50) or MUC-1 (1:200) overnight at 4°C in a dark wet chamber, followed by incubation with secondary antibodies (ZF-0316/ZF-0312; Zhongshan Golden Bridge, China) for 2 hours at room temperature. After washing with PBS, the nuclei were counterstained with DAPI (blue). Immunofluorescent images were captured by the Olympus IX51 (Olympus, Tokyo, Japan) confocal microscope. Experiments were repeated a minimum of three times.

### Clinicopathologic features

Tumor size was measured as the largest dimension grossly or microscopically. Lymph node status was obtained from pathologic report. The tumor grade and tumor-node-metastasis (TNM) stage were assigned according to the standard WHO classification [30]. IHC for ER, PR, and HER2 was re-evaluated at review of each case. Tumors with staining in >1% of tumor cell nuclei were defined as positive for ER and PR [32]. HER2 immunostaining was evaluated per the Dako HercepTest scoring system.

### Immunohistochemistry

IHC was performed using standard procedure [33] on properly prepared 4-μm tissue sections with the following primary antibodies: β1 integrin (1:300), Rac1 (1:1000), and MUC-1 (1:500). Negative controls were prepared by omitting the primary antibodies.

β1 integrin immunoreactivity was scored based on the percentage of positive tumor cells: 0: ≤ 10%; 1+: 11%–25%; 2+: 26%–50%; and 3+: > 50% [14]. Cases were assigned to high expression (2+ or 3+) or low expression (0 or 1+) groups, per the previously described method [34]. In Rac1 evaluation, cases were divided into three groups (0: 0%; 1+: 1%–33%; and 2+: ≥ 34%) and further classified as high expression (2+) or low expression (0 or 1+) [35]. MUC-1 reversed pattern was defined as the presence of complete linear reactivity on the outer surface of tumor cell clusters facing stroma (ie, polarity reversal) [36].

### Statistical analysis

Statistical analysis was carried out using SPSS 19.0. Differences among groups were analyzed using the Mann-Whitney *U* test. Correlations between two variables were evaluated using Spearman's rank correlation analysis. Survival curves for DFS and OS were constructed using the Kaplan-Meier method, and the differences between two groups were assessed using the log-rank test. Cox proportional hazards models were used to perform univariate and multivariable analysis. A two-tailed *P* < 0.05 was considered statistically significant.
